# Persistent localized activity in a two-population neural-field model with spatio-temporal external input

**DOI:** 10.1186/1471-2202-12-S1-P377

**Published:** 2011-07-18

**Authors:** Muhammad Yousaf, Gaute T Einevoll, Tom Tetzlaff, John Wyller

**Affiliations:** 1 Department of Mathematical Sciences, Norwegian University of Life Sciences, 1432 Ås, Norway

## 

**Figure 1:**
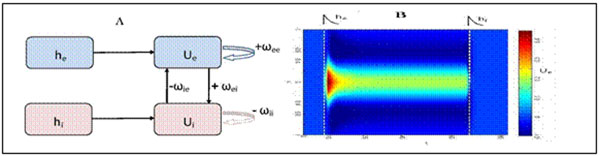
**A.** Sketch of the two population neural field model with excitatory (h_e_) and inhibitory (h_i_) external inputs. **B**. Persistent excitatory activity (Ue) switched on and off by a brief external input pulses. Inhibitory activity behaves in a similar way.

Persistent localized activity in neural networks has been suggested to serve as the neural substrate of short-term memory [1]. Neural-field models [2] provide a powerful tool to study the existence, uniqueness and stability of this type of activity. Bump solutions are often considered as an example of localized activity. In [3], it has been shown that a two-population neural-field model (Fig.1A) exhibits up to two coexisting bump-pair solutions in the presence of homogeneous external input (or in the absence of external input). Here, we show that the same system can generate up to four coexisting bump pairs if the external input is spatially localized.

In general, neural-field models are formulated in terms of Volterra-equation systems or systems of integro-differential equations. The dynamics of bump (pulse) solutions is often studied in a simplified framework by means of ordinary differential equations describing the time evolution of the pulse widths (Amari approach, see [4]).

It has been proven that in the absence of external input this simplified pulse-width system gives the correct prediction with respect to the linear stability of stationary solutions, with one notable exception. It is often conjectured that the Amari approach might correctly predict also nonlinear aspects of the dynamics (bifurcation analysis). Here, we show that the Amari approach fails in the presence of spatially localized external input: the criteria for linear-stability of bumps in the pulse-width system do not coincide with those obtained for the full system. Therefore, for localized external input the analysis must rely on the full system.

We determine fixed-point solutions and their stability analytically and illustrate the results by means of numerical simulations. Further, we numerically show that persistent localized activity in a two-population neural-field model can be switched on and off by means of brief external-input pulses localized in space in and time (see Fig 1B).
